# Gonorrhea and chlamydia prevalence in different anatomical sites among men who have sex with men: a cross-sectional study in Guangzhou, China

**DOI:** 10.1186/s12879-018-3579-6

**Published:** 2018-12-18

**Authors:** Li-Gang Yang, Xiao-Hui Zhang, Pei-Zhen Zhao, Zheng-Yu Chen, Wu-Jian Ke, Xu-Qi Ren, Liu-Yuan Wang, Wei-Ying Chen, Joseph D. Tucker

**Affiliations:** 1 Department of Sexually Transmitted Diseases, Dermatology Hospital, Southern Medical University, Guangzhou, 510091 China; 2 Guangdong Provincial Dermatology Hospital, Guangzhou, 510091 China; 3 Institute for Global Health and Infectious Diseases, University of North Carolina at Chapel Hill, Raleigh, USA; 4 Faculty of Infectious and Tropical Diseases, London School of Hygiene and Tropical Medicine, London, UK

**Keywords:** MSM, Chlamydia, Gonorrhea, Rectal, Pharyngeal

## Abstract

**Background:**

A high rectal and oropharyngeal sexually transmitted infection (STI) burden has been reported among men who have sex with men (MSM) in many regions, but little data exists on rectal and oropharyngeal STIs among MSM in China. The purpose of this study was to determine the prevalence of gonorrhea and chlamydia at different anatomic sites among MSM in Guangzhou, China.

**Methods:**

We recruited a cross-sectional sample of MSM in one Chinese city and collected detailed information about socio-demographic characteristics and sexual behaviors. Men had urine, rectal, and pharyngeal swab samples tested for gonorrhea and chlamydia using nucleic acid amplification tests (NAAT). Univariate and multivariate logistic regressions were used to evaluate factors associated with gonorrhea and chlamydia. Among men without any STI symptoms, we also examined the prevalence of gonorrhea and chlamydia by anatomical site.

**Results:**

We enrolled 463 men between January 2015 and March 2017. A total of 58/463 (12.5%) of men had gonorrhea and 84/463 (18.1%) had chlamydia. MSM with gonorrhea were more likely to have been recruited from the STI clinic (OR 3.41, 95% CI 1.94–5.99), living with HIV (OR 2.41, 95% CI 1.18–4.92), diagnosed had STI co-infection (OR 2.55, 95% CI 1.39–4.69). MSM with chlamydia were more likely to be students (OR 1.8, 95% CI 0.99–3.39). Most gonorrhea (34/58, 59%) and chlamydia (64/84, 76%) infections were not associated with STI symptoms.

**Conclusion:**

Asymptomatic gonorrhea and chlamydia infection were common in this sample of Chinese MSM. Further research is necessary to determine optimal STI screening programs.

## Background

Gonorrhea and chlamydia infections are the most common curable sexually transmitted infections (STIs). In 2012 there were an estimated 78 million (53–110 million) new gonorrhea infections and 131 million (100–166 million) chlamydia infections respectively, in 2012 [1]. Men who have sex with men (MSM) have particularly high rates of gonorrhea and chlamydia [2–7].

Extragenital gonorrhea and chlamydia are major public health problems among MSM. Compared with genital infections, rectal and pharyngeal gonorrhea and chlamydia infections are more likely to be asymptomatic [5] and can easily be missed without screening. Several high-income countries recommend regular screening of extragenital sites (rectum and pharynx) among sexually active MSM [8–10]. World Health Organization MSM preventive guidelines also support gonorrhea and chlamydia screening among MSM with a higher burden of asymptomatic disease [11]. This suggests the need for research to understand the burden of extragenital gonorrhea and chlamydia among MSM in different settings.

Despite high HIV prevalence and frequent condomless sex among MSM in China, [12–18] there are limited data on MSM extragenital gonorrhea and chlamydia. [19, 20] Better understanding the burden of rectal and pharyngeal gonorrhea and chlamydia can provide useful information for research and screening pilots. We undertook this study in Guangzhou, China. Guangzhou is a provincial capital city in south China with a concentrated HIV infection in MSM [21] and a strong STI network. The purpose of this study was to determine the prevalence of gonorrhea and chlamydia at different anatomic sites among MSM in Guangzhou, China.

## Methods

### Study population and procedure

We recruited a cross-sectional convenience sample of MSM between January 2015 and March 2017. Enrollment criteria included being male, currently living in Guangzhou, being at least 18 years of age, self-identifying as gay or bisexual, and receiving no antibiotics in the past month.

We recruited patients from two clinics at the Dermatology Hospital of Southern Medical University, a tertiary care center with experience serving MSM. The first was an STI clinic run by the hospital, through which any patient who had STI complaints or concerns could request to see a doctor. Patients meeting enrollment criteria who agreed to participate were asked to join the research study. The second was a weekend community clinic sponsored by the Zhitong Guangzhou LGBT (lesbian, gay, bisexual, and transgender) Center, an MSM community organization. This community clinic provides free HIV and syphilis testing services for MSM. During this study, Zhitong also advertised our project on their website and social media account.

### Data collection

At enrollment, we collected baseline socio-demographic information, sexual behavior, and STI history. Information about symptoms was collected as a single item. If men had symptoms consistent with an STI, this was noted. There was no effort to attribute symptoms to a particular pathogen in cases of co-infection. All participants underwent physical examination, and any abnormal findings (i.e. warts) were recorded. Urine, rectal and pharyngeal swab samples were collected for gonorrhea and chlamydia testing. Venous blood samples were collected for HIV and syphilis testing. Men with positive tests were treated according to Chinese STI clinical management guidelines.

### Laboratory methods

Oropharyngeal, rectal, and urine specimens were tested for gonorrhea and chlamydia using the Cobas 4800 system (Roche Moleculer Systems, Inc. New Jersey, USA). Blood samples were tested for syphilis using a toluidine red unheated serum test (TRUST, Rongsheng Bio-technology Limited Corporation, Shanghai, China) and *Treponema pallidum* particle agglutination test (TPPA, Fujirebio Inc., Japan). Blood samples were tested for HIV using two antibody tests - a rapid HIV antibody test (Wantai, Beijing, China) and a second antibody test (Abon Biopharm, Hangzhou, China). If both were positive, another blood sample was collected for Western blot confirmation (MP Biomedical, Singapore).

Men were considered infected with gonorrhea and chlamydia if respective tests were positive. Men with positive TRUST and TPPA tests were considered to have syphilis infection, unless a documented history of previously treated syphilis was available. Men with positive HIV screening and confirmatory tests were considered to have HIV infection. Anogenital warts and anogenital herpes were diagnosed according to the diagnostic criteria for China’s notifiable diseases reporting system (Version 2008). These diagnoses are mainly based on exposure history and consistent clinical findings.

### Data analysis

We described socio-demographic characteristics, sexual risk behaviors, and STI (by anatomic site) among men. We compared men presenting to the STI clinic and men presenting to the MSM community clinic. Chi-square tests were used to compare gonorrhea/chlamydia prevalence between the STI clinic and MSM community groups. Univariate and multivariate logistic regressions were used to evaluate factors associated gonorrhea and chlamydia, respectively. We also calculated the frequency of men with gonorrhea and chlamydia who were asymptomatic. All data were analyzed using SAS 9.2 (SAS, Cary, USA).

### Ethics, consent and permissions

Ethical approval for this study was obtained from the Science Research Ethical Committee of the Dermatology Hospital of Southern Medical University (GDDHLS-201502). Written informed consent was waived because the risk associated with participating in this study was deemed minimal and involved no procedures requiring consent outside of the context of participating in the study. This was approved by the Chinese IRB. It is also in accordance with DHHS (45 CFR 46.117). Verbal consent was obtained before participants were enrolled.

## Results

We recruited 463 MSM to join the study. One-hundred and fifty-three men were from the STI clinic and 310 men were from the MSM community clinic. Most men were young, Han ethnicity, unmarried, non-students, and had a higher education level. Socio-demographic characteristics are shown in Table 1.

**Table 1:** Demographic and behavioral characteristics of participants from the hospital STI clinic and the community-based MSM clinic (*n* = 463), 2015–2017

	STI Clinic n (frequency)	Community clinic n (frequency)	*P-value*
Age(years)			0.159
< 25	50/153 (32.7%)	128/310 (41.3%)	
25~35	68/153 (44.4%)	113/310 (36.4%)	
> 35	35/153 (22.9%)	69/310 (22.3%)	
Ethnicity			0.088
Han	146/153 (95.4%)	282/310 (91.0%)	
Others	7/153 (4.6%)	28/310 (9.0%)	
Marital status			< 0.0001
Unmarried	101/153 (66.0%)	265/310 (86.6%)	
Married	50/153 (32.7%)	35/310 (11.4%)	
Other	2/153 (1.3%)	6/310 (2.0%)	
Student			0.049
Yes	14/153 (9.2%)	49/310 (15.8%)	
No	139/153 (90.8%)	261/310 (84.2%)	
Local residence time			0.854
0–6 months	22/153 (14.4%)	44/310 (14.5%)	
6–12 months	9/153 (5.9%)	22/310 (7.3%)	
Over 1 year	122/153 (79.7%)	237/310 (78.2%)	
Education level			0.065
Junior middle school and below	22/153 (14.4%)	24/310 (7.8%)	
Senior middle school	22/153 (14.4%)	41/310 (13.2%)	
Senior middle school above	109/153 (71.2%)	245 (79.0%)	
Any STI related symptoms			< 0.001
Yes	49/153 (32.0%)	31/310 (10.0%)	
No	104/153 (68.0%)	279/310 (90.0%)	
Condom use in the last 6 months			< 0.001
Any condom less sex	141/153 (92.8)	305/310 (99.3%)	
Consistent condom use	11/153 (7.2%)	2/310 (0.7%)	

**Fig. 1:**
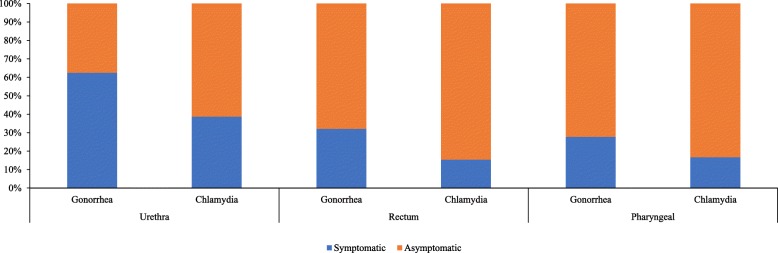
Proportion of symptomatic and asymptomatic urethral, rectal and pharyngeal chlamydia and gonococcal infections

Among 463 men, a total of 58 (12.5%) men had gonorrhea infection. The gonorrhea prevalence at urethral, rectal, and pharyngeal sites was 5.2% (24/463), 6.1% (28/463), and 3.9% (18/463), respectively (Table 2). Among all men with gonorrhea, most men (34/58, 59%) did not have STI symptoms. MSM with asymptomatic extragenital gonorrhea were common (Fig. 1). MSM with gonorrhea were more likely to have been recruited from the STI clinic (OR 3.41, 95% CI 1.94–5.99), living with HIV (OR 2.41, 95% CI 1.18–4.92), and diagnosed with STI co-infection (OR 2.55, 95% CI 1.39–4.69) (Table 3).

**Table 2:** Gonorrhea and chlamydia infection by anatomic sites among 463 MSM in an STI clinic and a community-based clinic in Guangzhou China, 2015–2017

Variables	STI clinic n (frequency)	Community clinic n (frequency)	Total n (frequency)	P*
Gonorrhea	34/153(22.2%)	24/310 (7.7%)	58/463 (12.5%)	< 0.001
Urethral	22/153 (14.4%)	2/310 (0.7%)	24/463 (5.2%)	< 0.001
Rectal	11/153 (7.2%)	17/310 (5.5%)	28/463 (6.1%)	0.469
Pharyngeal	6/153 (3.9%)	12/310 (3.9%)	18/463 (3.9%)	0.979
Chlamydia	29/153 (19.0%)	55/310(17.7%)	84/463 (18.1%)	0.750
Urethral	18/153 (11.8%)	13/310 (4.2%)	31/463 (6.7%)	0.002
Rectal	10/153 (6.5%)	42/310 (13.6%)	52/463 (11.2%)	0.025
Pharyngeal	1/153 (0.7%)	5/310 (1.6%)	6/463 (1.3%)	0.391
HIV	20/153 (13.1%)	40/310 (12.9%)	60/463 (13.0%)	0.959
^a^Other STIs	49/153 (32.0%)	35/310 (11.3%)	84/463 (18.1%)	< 0.001

*Chi-squared test

^a^Other STIs refers to syphilis, ano-genital warts and herpes

26 participants were co-infected with gonorrhea and chlamydia

**Table 3:** Factors associated with gonorrhea and chlamydia infections among 463 MSM in Guangzhou China, 2015–2017

	gonorrhea(%)	*OR (95%CI)*	*P*	AOR	chlamydia(%)	*OR(95%CI)*	*P*	AOR
Age(years)								
< 25	26/178 (14.6%)	1.81 (0.81,4.02)	0.148	–	37/178 (20.8%)	1.34 (0.71,2.53)	0.362	
25~35	23/181 (12.7%)	1.54 (0.68,3.46)	0.299	–	30/181 (16.6%)	1.02 (0.53,1.95)	0.960	
> 35	9/104 (8.7%)	1.00		–	17/104 (16.4%)	1.00		
Ethnicity								
Han	52/428 (12.2%)	0.67(0.27,1.69)	0.394	–	76/428 (17.8%)	0.73(0.32,1.67)	0.453	
Others	6/35 (17.1%)	1.00		–	8/35 (22.9%)	1.00		
Marital status								
Unmarried	45/366 (12.3%)	0.98(0.12,8.16)	0.986	–	63/366 (17.2%)	0.62(0.12,3.16)	0.568	
Married	12/85 (14.1%)	1.15(0.13,10.20)	0.899	–	17/85 (20.0%)	0.75(0.14,4.05)	0.737	
Other	1/8 (12.5%)	1.00		–	2/8 (25.0%)	1.00		
Student								
Yes	11/63 (17.5%)	1.59(0.78,3.26)	0.206	–	17/63 (27.0%)	1.84(0.99,3.39)	0.053	
No	47/400 (11.8%)	1.00		–	67/400 (16.8%)	1.00		
Local residence time								
0–6 months	12/66 (18.2%)	1.59(0.79,3.21)	0.194	–	12/66 (18.2%)	0.99(0.50,1.95)	0.969	
6–12 months	2/31(6.5%%)	0.49(0.11,2.14)	0.346	–	4/31 (12.9%)	0.66(0.22,1.94)	0.449	
Over 1 year	44/359 (12.3%)	1.00		–	66/359 (18.4%)	1.00		
Education level								
Junior middle school and below	6/46 (13.0%)	1.03(0.41,2.57)	0.949	–	7/46 (15.2%)	0.74(0.32,1.73)	0.488	
Senior middle school	7/63 (11.1%)	0.86(0.39,2.00)	0.723		8/63 (12.7%)	0.60(0.27,1.32)	0.204	
Senior middle school above	45/354 (12.7%)	1.00			69/354 (19.5%)	1.00		
Recruited from								
STI clinic	34/153 (22.2%)	3.41(1.94,5.99)	< 0.01	2.71(1.49,4.96)	29/153 (19.0%)	1.08(0.66,1.79)	0.750	–
Community clinic	24/310 (7.7%)	1.00		1.00	55/310 (17.7%)	1.00		–
Any STI related symptoms								
Yes	24/80 (30.0%)	4.40(2.43,7.97)	< 0.01	2.89(1.53,5.47)	20/80 (25.0%)	1.66(0.94,2.95)	0.082	–
No	34/383 (8.9%)	1.00		1.00	64/383 (16.7%)	1.00		–
Condom use in the last 6 months								
Any condom less sex	56/446 (12.6%)	0.79(0.17,3.66)	0.763	–	82/446 (18.4%)	1.23(0.27,5.70)		–
Consistent condom use	2/13 (15.4%)	1.00		–	2/13 (15.4%)	1.00		–
No. of sex partners in last 6 months	–	1.04(0.94,1.15)	0.424	–	–	1.09(1.01,1,18)	0.0495	1.09(1.01,1,18)
HIV positive								
Yes	15/60 (25.0%)	2.79(1.44,5.42)	0.0025	2.41(1.18,4.92)	15/60 (25.0%)	1.61(0.85,3.06)	0.1424	–
No	43/403 (10.7%)	1.00		1.00	69/403 (17.1%)	1.00		–
Other STIs^#^								
Yes	19/84 (22.6%)	2.55(1.39,4.69)	0.0026	–	18/84 (21.4%)	1.29(0.72,2.32)	0.3885	–
No	39/379 (10.3%)	1.00		–	66/379 (17.4%)	1.00		–

#: other STIs refers to syphilis, ano-genital warts and ano-genital herpes

A total of 84 men (18.1%) had chlamydia infection at any site. The chlamydia prevalence at the urethral, rectal and oropharyngeal sites was 6.7% (31/463), 11.2% (52/463), and 1.3% (6/463), respectively. Among all men with chlamydia, most men (64/84, 76%) did not have STI symptoms. 61.3% of urethral infections, 83.3% of rectal infections and 83.3% of pharyngeal infections were asymptomatic (Fig. 1). There was a trend towards MSM with chlamydia being more likely to be students (OR 1.8, 95% CI 0.99–3.39).

The chlamydia and gonorrhea coinfection rate was 5.6% (*n* = 26). Sixty men (13.0%) were living with HIV infection. Eighty-four men (18.1%) had other STIs, including syphilis, anogenital wards, or herpes.

## Discussion

Our study evaluated the prevalence of urethral, anal, and pharyngeal gonorrhea and chlamydia among MSM in Guangzhou, China. This study expands the limited literature on MSM extragenital gonorrhea and chlamydia prevalence in an low and middle income country context. This study also compares two different populations of MSM within the same city, providing insight about risk in these groups.

The overall chlamydia prevalence was 18.1%, and the gonorrhea prevalence was 12.5%. The burden of chlamydia and gonorrhea are similar to findings from other Chinese studies in Shenzhen [19] and Kunming [20]. Over half of all STIs were asymptomatic, consistent with other studies. Given that World Health Organization Guidelines recommend MSM screening for asymptomatic urethral and rectal gonorrhea/chlamydia if prevalence is greater than 1–2% [11], our data suggest that screening would be indicated in this population.

We found that the majority of MSM STI infections were asymptomatic. This is consistent with a previous study from the United States [5]. This trend is more pronounced for rectal and pharyngeal infections [22]. The high burden of extra-genital STIs among MSM may help researchers and policy makers making decisions about routine screening guidelines.

Our data suggest that more MSM with symptoms seek care at the STI clinic compared to the MSM community clinic. This is consistent with the STI literature on MSM seeking care at STI clinics compared to community clinics [5]. Asymptomatic infections were especially common in the MSM community clinic. This suggests that asymptomatic screening programs may be more appropriate for MSM community clinics and related settings.

Our study has implications for STI research priorities among MSM. Better understanding the burden of asymptomatic extragenital STIs may be helpful in designing prospective screening studies or modelling research. Despite the fact that many guidelines recommend periodic STI screening among MSM, there are also barriers to screening (cost. Feasibility, logistics). The US Preventive Service Task Force found insufficient evidence to recommend gonorrhea and chlamydia testing among men when it last reviewed the evidence [23]. Accurate assessment of asymptomatic extragenital MSM STIs will also be helpful for the development of local policies and practice guidelines.

Our study has several limitations. First, our study was limited to two clinics taking place in the same hospital in Guangzhou. This study is not representative of MSM in China and caution should be used in making generalizations to other settings. Second, the study was cross-sectional in nature, so we cannot draw conclusions regarding causality. Third, although we asked patients about their symptoms, the main purpose of the survey instrument was not to better understand co-existing symptoms. In addition, symptoms may not have been related to an STI. Pharyngeal symptoms have a wide differential diagnosis, so our estimate of asymptomatic cases of pharyngeal chlamydia and gonorrhea are likely underestimates.

## Conclusions

Our findings show that chlamydia and gonorrhea prevalence are high among Chinese MSM. Enhanced screening and treatment of rectal chlamydia and gonorrhea among MSM is necessary to improve sexual health.

## Abbreviations

DHHS: Department of Health and Human Services
LGBT: Lesbian, gay, bisexual, and transgender
MSM: Men who have sex with men
NAAT: Nucleic acid amplification tests
STI: Sexually transmitted infection
STIs: Sexually transmitted infections
TPPA: Treponema pallidum particle agglutination test
TRUST: Toluidine red unheated serum test
